# Reproducibility of prediction models in health services research

**DOI:** 10.1186/s13104-022-06082-4

**Published:** 2022-06-11

**Authors:** Lazaros Belbasis, Orestis A. Panagiotou

**Affiliations:** 1 Meta-Research Innovation Center Berlin, QUEST Center, Berlin Institute of Health, Charité – Universitätsmedizin Berlin, Berlin, Germany; 2 Center for Evidence Synthesis in Health, School of Public Health, Brown University, Providence, RI USA; 3 Department of Health Services, Policy and Practice, School of Public Health, Brown University, Providence, RI USA; 4 Department of Epidemiology, School of Public Health, Brown University, Providence, RI USA

**Keywords:** Health services research, Machine learning, Open Science, Prediction modelling, Reproducibility, Research Methodology, Transparency

## Abstract

The field of health services research studies the health care system by examining outcomes relevant to patients and clinicians but also health economists and policy makers. Such outcomes often include health care spending, and utilization of care services. Building accurate prediction models using reproducible research practices for health services research is important for evidence-based decision making. Several systematic reviews have summarized prediction models for outcomes relevant to health services research, but these systematic reviews do not present a thorough assessment of reproducibility and research quality of the prediction modelling studies. In the present commentary, we discuss how recent advances in prediction modelling in other medical fields can be applied to health services research. We also describe the current status of prediction modelling in health services research, and we summarize available methodological guidance for the development, update, external validation and systematic appraisal of prediction models.

## Introduction

Health services research is a multidisciplinary field that studies the health care system, including access to and delivery of care; the quality of the care provided to patients; the costs of care for patients, health systems, and payers; and ultimately the impact of care on health outcomes and well-being [1]. Data sources in health services research often differ from the traditional epidemiological investigations that prospectively or retrospectively collect data through active recruitment of participants based on a priori specified research questions [2]. Indeed, health services studies heavily rely on data that are routinely collected for purposes other than research, including health care billing claims, registry data, or electronic health records [1, 3]. Outcomes frequently examined by health services researchers include health care spending, and utilization of care services (e.g., hospital admission or readmission, admission to intensive care unit, length of hospitalization, or emergency department visit).

Making accurate predictions of these outcomes is crucial from the perspective of patients, clinicians, health economists, and policy makers. On the basis of prediction horizon, prediction models are classified into two categories: (a) diagnostic models (absence of a time horizon) and (b) prognostic models (presence of a time horizon). During the last decade, there has been an intensified discussion about the reproducibility of statistical methods for predicting outcomes in medicine, and more recently this discussion has expanded to prediction modelling using machine learning techniques [4]. Ensuring reproducible prediction models in health services research is critical for the deployment of these models in real-world settings to inform clinical and health policy decision-making.

In this commentary, we discuss recent advances in prediction modelling in fields such as clinical medicine that are relevant to ensuring reproducible models in health services research. While diagnostic modelling is common in health services studies (e.g., when developing algorithms for the accurate ascertainment of disease status from billing codes in administrative data), we focus here on prognostic models that predict a future outcome of interest over a time horizon, because these types of questions frequently concern health services problems. We present what is already known about prediction modelling in other medical fields, describe the current status of prediction modelling in health services research, and present recommendations and guidance to improve current research practices in this field.

## Main text

### Reproducibility and transparency in prediction modelling

Reproducibility, transparency, and openness are three interconnected concepts that are readily recognized as vital features of science [5–7]. Reproducibility is the ability of independent researchers to obtain the same (or similar) results when repeating an experiment or test, and it is considered a hallmark of high-quality science [8, 9]. Irreproducible research can occur because of practices applied in one or more steps involving study design, data quality, statistical analysis or study reporting [8]. Of direct relevance to prediction modelling (especially when machine learning methods are used) is computational reproducibility, which refers to the ability to repeat an analysis of a given dataset and obtain sufficiently similar results [10, 11]. It requires having available the complete analytical environment, including software, properly documented full source code, and the original data [10]. Ideally, the user and/or researcher should be able to inspect, modify and apply the code under modified parameter settings to reproduce the results and explore the robustness of the algorithm to the values of its parameters. In recent years, platforms designed for the development of software, such as GitHub, have been adopted by the scientific community as ways to distribute the code including many health services projects [10].

Transparency is another important component of high-quality research. Two major transparency measures are registration and pre-published protocols, which can reduce the selective reporting of prediction models. Although their importance in the context of randomized clinical trials is widely accepted and strongly promoted, their importance in prediction modelling research is not widely acknowledged [12]. Openness, a term including data and code sharing, is also a key indicator of high-quality research, but it remains an uncommon practice in prediction modelling research [12]. Promoting data and code sharing is expected to increase the number of external validation efforts and individual-participant data meta-analyses in prediction modelling for health services research. These processes can be enhanced by formalizing the data management and data sharing processes using the FAIR guiding principles for scientific data management and stewardship [13]. This document presents guidelines to improve the Findability, Accessibility, Interoperability, and Reuse of data.

However, it should be acknowledged that data sharing in the context of health services research may be challenging. The main reason is that many routinely collected health data are provided to researchers for scientific purposes only upon approval by the entity that generates them (e.g., health insurer, health system etc.) under strict agreements to protect patient confidentiality and privacy [14]. Although these agreements often preclude further data sharing among researchers, data sharing could be facilitated through the development of large scientific consortia which have been successful in other research fields such as genetic epidemiology [15, 16].

### Experience from prediction modeling in other research fields

Several large-scale systematic reviews of prediction models in clinical medicine have evaluated the quality of clinical prediction models and their potential to yield unbiased predictions. A summary of multiple risk-of-bias assessments examining more than 2000 models using PROBAST (i.e., a risk-of-bias assessment tool) showed that (a) two thirds of them have high risk of bias based on their statistical analysis, (b) one third of them had high risk of bias based on their outcome definition and ascertainment and (c) a quarter of them had high risk of bias based on how participants were selected [17]. Moreover, one of the largest systematic reviews of prediction models examined more than 400 models for outcome prediction in patients with chronic obstructive pulmonary disease [18]. The vast majority of the examined prediction models did not report the full model equation or any other form of model presentation. This is an important caveat of prediction models, because absence of any model presentation renders any effort to assess the reproducibility of a prediction model impossible and further diminishes the opportunity to deploy prediction models in routine settings, even if they have outstanding performance.

There have also been several assessments of machine learning models in areas outside health services research. Machine learning is a large family of statistical techniques with a rapidly increasing use in prediction modelling, especially in the field of health services research [1, 19]. Yet many prediction models based on machine learning methods have important limitations. For example, their adherence to reporting guidelines is often suboptimal thereby reducing their potential to be reproduced and deployed in independent studies [20]. Additionally, a risk-of-bias assessment of multiple prediction models using supervised machine learning showed that almost 90% of the models were at high risk of bias [21]. Moreover, the handling of missing data is rarely reported, and when authors deal with missing data, they often poorly report the relevant methodological details [22]. These issues not only threaten the validity of statistical estimates but also make these models hard to reproduce. It is, therefore, important that health services researchers recognize these issues in advance and take proactive steps to ensure that prediction models addressing health services questions are not subject to similar limitations.

### Prediction modelling in health services research

Numerous systematic reviews for prediction models have been published, and some of them focus on predicting outcomes relevant to health services research. For example, there are systematic reviews focused on prediction models for re-admission after an index hospitalization [23, 24], emergency hospital admission [25], length of hospital stay [26, 27], length of stay in the intensive care unit [28], and health care costs [29]. These systematic reviews summarize many prediction models, but their focus is on the data sources used, the predictors used and the model performance without providing a thorough assessment of reproducibility, transparency, and study quality.

Moreover, prediction models for outcomes relevant to health services research are often included in systematic reviews focusing on patients with a specific disease. For example, in a systematic review for patients with chronic obstructive pulmonary disease, 65 prediction models were identified for outcomes relevant to health services research (i.e., hospital admission, ICU admission, readmission after an index hospitalization, length of stay, and health care costs) [18]. However, there is a need for more systematic assessments of prediction models focusing exclusively on outcomes relevant to health services research. These systematic reviews could be used to draw important observations and recommendations to improve the development and validation of prediction models in this field. Of note, systematic reviews of prediction models should also adhere to the principles of open science to the extent possible. A starting point is pre-registration through relevant repositories or even journals that publish protocols of systematic reviews. For example, we recently published a protocol of a systematic review of multivariable models for prediction of health care spending using machine learning by following all the relevant frameworks and methodological guidance [30]. We hope that this research practice can become more prevalent in the near future.

### Existing guidance for prediction models

A critical step in improving the reproducibility and research quality in prediction modelling for health services research is to systematically map the current research practices in this field. Through this process the issues contributing to irreproducibility and poor reporting of prediction models will be identified. However, to our knowledge, existing systematic reviews on prediction models for outcomes relevant to health services research have not performed a thorough assessment of prediction models.

Various frameworks are available for performing systematic reviews for prediction models, and we recommend that researchers follow them when conducting systematic reviews [31]. Also, the PRISMA statement is a general framework that was developed to guide any systematic review and meta-analysis in biomedical literature [32, 33]. To support the conduct of systematic reviews of prediction models, there is a validated search algorithm for prediction modelling studies in PubMed [34], and a guidance on how to construct a data extraction form [35]. Both these items can make the systematic review process more efficient, reproducible, and transparent. In addition, PROBAST, a risk-of-bias assessment tool for prediction modelling studies, can help contextualize biases arising from the selection of participants, the ascertainment of the outcome, the handling of predictors, and the statistical methods used for prediction [36, 37]. An extension of this tool (PROBAST-AI) for the assessment of prediction modelling studies using machine learning approaches is currently under development [38].

Researchers should also consider the life cycle of prediction modelling research, as it was previously described, before developing a new prediction model [39–42]. Based on the PROGRESS framework [39], the researchers should avoid developing new prediction models from scratch without ensuring that existing models are inadequate. Instead, when prediction models exist, they should aim to update them to improve their predictive performance and externally validate them to examine their generalizability in other populations. Moreover, before the deployment of prediction models in clinical practice or their use in decision-making, impact studies should be designed to assess their impact in real world settings [42].

The development, update and external validation of prediction models in health services research could be improved by following guidance that was developed during the last decade for clinical prediction models. Health services researchers building a prediction model should follow the TRIPOD statement, which is a set of recommendations for the reporting of studies developing, validating or updating a prediction model and is endorsed by many journals [43, 44]. Although the TRIPOD statement was developed for traditional (parametric) statistical models, there is an ongoing process of developing the TRIPOD-AI statement, which will provide recommendations exclusively for machine learning models [38]. Also, there is additional guidance explaining how the prediction models should be presented [45].

Some additional guidance has been developed for prediction models using machine learning approaches. The MI-CLAIM checklist was developed to improve transparent reporting of machine learning algorithms in medicine, and it has similarities with TRIPOD statement [46]. Also, there is an additional framework on transparency, reproducibility, ethics, and effectiveness in machine learning applications for health [47]. Some standards for the computational reproducibility of machine learning models have been proposed, based on data, model and code publication, programming best practices and workflow automation [48, 49].

## Outlook

Adhering to reproducible and transparent research practices when developing and employing a prediction model in health services research is important for the design of efficient health systems and health delivery programs, and the improvement in patients’ outcomes. In this commentary, we summarize available frameworks and guidelines to develop, externally validate, update, and systematically review prediction models, and we discuss potential implications in health services research. These frameworks and approaches to reproducible prediction modelling that we discuss here require involvement from multiple stakeholders beyond individual researchers. Such stakeholders involve journal editors, peer-reviewers, funding bodies and universities, who can play a critical role in promoting, incentivizing and rewarding reproducible and transparent research practices.

## Data Availability

Not applicable.

## Abbreviations

FAIR: Findability, accessibility, interoperability, and reuse
ICU: Intensive care unit
MI-CLAIM: Minimum information about clinical artificial intelligence modeling
PRISMA: Preferred reporting items for systematic reviews and meta-analyses
PROBAST: Prediction model risk of bias assessment tool
PROBAST-AI: Prediction model risk of bias assessment tool-artificial intelligence
TRIPOD: Transparent reporting of a multivariable prediction model for individual prognosis or diagnosis
TRIPOD-AI: Transparent reporting of a multivariable prediction model for individual prognosis or diagnosis-artificial intelligence
